# Health equity innovation in precision medicine: Current challenges and future directions

**DOI:** 10.3389/fpubh.2023.1119736

**Published:** 2023-02-15

**Authors:** Marcia G. Ory, Omolola E. Adepoju, Kenneth S. Ramos, Patrick S. Silva, Deborah Vollmer Dahlke

**Affiliations:** ^1^Department of Environmental and Occupational Health, School of Public Health, Texas A&M University, College Station, TX, United States; ^2^Center for Community Health and Aging, Texas A&M School of Public Health, College Station, TX, United States; ^3^Department of Health Systems and Population Health Sciences, Tilman J. Fertitta Family College of Medicine, University of Houston, Houston, TX, United States; ^4^Humana Institute, Tilman J. Fertitta Family College of Medicine, University of Houston, Houston, TX, United States; ^5^Institute of Biosciences and Technology, Texas A&M Health Science Center, Houston, TX, United States; ^6^Texas A&M Health Science Center, Houston, TX, United States; ^7^DVD Associates, LLC, Austin, TX, United States

**Keywords:** health equity, precision medicine, idealized clinicogenomic registry, community-centered approach, aging

## Introduction

Health equity is a global concern in health care. While a US Healthy People 2030 goal is to make health care more accessible to Americans, major gaps remain based on socioeconomic status, race, ethnicity, and geography (1). Whether equity challenges will increase or ameliorate as personalized medicine and genomics usher in a new wave of diagnostics and treatments remains unknown (2). A recent Health Equity Innovation Summit on Future Directions in Personalized Medicine, Genomics, and Clinical Care hosted by Texas A&M Health (https://www.healthequityinnovation.org/) explored this landscape—its challenges and solutions. Featuring healthcare expert panels and speakers that included patient groups, care providers, policymakers, technology companies, and academics, the Innovation Summit discussed policy and practice actions to build networks and collaborations. Recurring themes in the Summit addressed determinates of healthcare, including access to clinical trials and cutting-edge treatments. The Summit discussants provided a framework to inform future directions in precision medicine, genomics, clinical research, and healthy aging –and the policies that can impede or catalyze these critical dimensions of healthcare. Voice of the Patient strategies were discussed as a means for providers and investigators to appreciate nuance in niche populations generally, and for these populations to appreciate their opportunity in improving data driven healthcare models and their own health (3).

## The promises and challenges of precision medicine

The field of precision medicine has long been considered the future of healthcare. Introduced in 2011 as biomedical research, the field gained exponential exposure and praise, leading to the creation of the Precision Medicine Initiative in 2015 under the Obama administration with a $215 million investment in the nation's 2016 Budget (4, 5). Rather than the traditional one-size fits all approach to managing disease, precision medicine harnesses the power of big data and advances in biotechnology to generate more precise ways of treating disease. Through the partnered use of precision medicine and genomics, some treatments have showcased a reduction of mortality and morbidity in millions of patients with conditions, such as familial hypercholesterolemia, oncology, and psychiatric disorders. Overall, the field has paved its way through new discoveries, FDA-approved treatments, and risk stratification paradigms (6). The tools of precision medicine and predictive data sciences are crucial to shifts in practice guidelines, including preventative medicine and reimbursement policies, that can fulfill the promise of healthy aging. While many ongoing breakthroughs are geared toward treating disease, the propensity of this field in preventing rather than managing disease remains in its infancy.

Disease prevention across the life course remains the hallmark of health promotion and population health. Unfortunately, underrepresented minority (URM) communities in the US often present with greater risks, and at later stages that predispose community members to diseases and worse health outcomes, respectively. Perpetuated by a myriad of factors, including long-standing mistrust of the healthcare system, geographical distance from care, language barriers, and fear of encountering implicit bias and stereotyping during care, among others, URM communities are often at greater risk for sub-optimal health outcomes. For these populations, traditional medical approaches that focus on the disease incidence and management may produce mixed results—success, no change, or adverse effects following disease incidence— as they fail to account for underlying social, cultural, environmental, and lifestyle factors that contribute to health. Studies have shown that these inconsistent outcomes may also be linked to a low representation of URM in genomic registries and other clinical trials (7). These earlier studies argue that lower rates of clinical trial participation from URM groups shape future treatments so that meaningful treatment options for these communities are unequally distributed (8). This is further concerning as Turner et al. report that about 80 percent of the US clinical trials' pool consists of non-Hispanic white populations suggesting that other population groups might experience fewer benefits from biomedical research due to poor representation and barriers to healthcare access (9, 10).

This has direct implications for the field of precision medicine in that groups less likely to receive precision medicine treatments are racial minority and ethnic groups, medically underserved urban and rural communities, uninsured and underinsured, as well as those with lower education and income (11). More so, while precision medicine commonly uses genomic data for patient treatment plans, genome-wide association studies (GWAS) rarely test the relationship between complex genetic traits and environmental exposure (8). We posit that precision medicine for prevention and treatment holds promise in advancing health, particularly for medically underserved urban and rural groups, many of whom identify as belonging to one or more underrepresented groups and with varying degrees of genetic admixture (e.g., biogeographical ancestry analysis), an area in genomics that remains largely unexplored. Genetic variants of low frequency are likely disproportionately important in disease (12).

Frequent confusion within the field of precision medicine stems from the absence of defined population categories leading to inconsistent misrepresentation or classification of underrepresented communities in clinical trials (13). For example, grouping all racialized groups together often creates challenges with translating study findings into the real-world setting where racialized groups are far from a homogenous population. In recent years, ethnicity determined through genomic analysis has been proposed as a more precise approach to contextualize disparities rather than the social construct of race (14). For example, the use of genetic patterns, including variations of drug metabolism and drug targets, indicates that there are issues in representing human population genetic structures in evaluating drug safety and efficiency and relating this structure to drug response. Commonly used ethnic labels are both insufficient and inaccurate representations of inferred genetic clusters, with the possible result that drug-metabolizing clusters would differ significantly (15). The potential solution to this is increased clinical implementation of pharmacogenomics based on the increased inclusion of underrepresented groups to guide drug therapy.

It is also important to acknowledge and address the contentious relationship between URM and clinical trials as long-term research endeavors often have negative connotations long past their initial purpose. One of the most notable examples for African American communities is the USPHS Syphilis Study at Tuskegee which is often viewed as a major source of healthcare mistrust in the African American community (16). Conducted from 1932 to 1972, this infamous natural history study neglected informed consent, denied available treatment, and produced meager benefits compared to human subjects' benefits. Other remembrances include the role of Dr. J. Marion Sims, the father of gynecology, who performed surgeries on “slave women,” experimenting on them without the use of anesthetics to perfect his technique to repair vesicovaginal fistulas. Further, there is collective memory about “night doctors,” who would steal cadavers of African American individuals to learn more about human body processes (17). Other studies have reported fear and mistrust in Hispanic communities as contributing to poor patient engagement. For example, Davis et al. (18) reported that Hispanics lacked trust in medical professionals and “feared being a guinea pig,” whereas those less educated cited a fear of being embarrassed during cancer screenings. Rodriguez-Madera et al. (19) noted that for Puerto-Ricans, there is a history of mistrust in the government, emanating from resentment over its perceived sense of alienation and “rooted in a longer history of political ineptitude.”

## Toward community-centered approaches

Trust issues continue to permeate modern-day healthcare. Addressing these barriers requires an understanding of these issues and, in turn, the ability to use that knowledge to develop community-centered approaches to reach these populations. One approach to address this is the Community Health Workers (CHWs) or promotores model, in which lay health workers serve on the frontlines to facilitate access to services through education and connections to social services and resources (20). Within the US healthcare system, CHWs often act as a bridge between a patient's healthcare provider and the patient themselves and work to improve this relationship by tailoring care specifically to the patient's needs in a manner that is culturally competent and appropriate to the patient. This approach improves engagement with its patient-centered responsiveness, rather than a generalized response that often misses its mark (21).

Other community-centered approaches include the use of community champions who are from the community, look like the community, speak the same language as the people in the community, and share relevant contextual experiences. It is also important to engage the community at the beginning of clinical trials and consider their input in the co-design and development of initiatives that will apply to them. For example, the National Institutes of Health has launched the “All of Us” research initiative that includes several innovations to address health equity, including dedicated efforts to reach, engage and retain diverse populations, cover many different geographies and settings, coordinate different data sets, and make community engagement a central outreach approach (22). Similarly, the National Human Genome Research Institute has launched a long-term partnership with Alaskan Native tribes to “overcome logistical and communication barriers in the hope of encouraging their involvement” in clinical research (23).

Continuous engagement creates an atmosphere of transparency which, in turn, builds trust and gives patient agency and fosters the translation of evidence-based practice from “bench to bedside.” Processes that can be used to reach this point include ongoing participation in community-led events (in place of one-time partnerships), layperson explanation of precision medicine, and data collection and usage, all while setting clear parameters for compensation, and benefits to the individual and the community. Such efforts can come to fruition through multisectoral collaborations that involve key stakeholders from diverse settings including universities, industry, communities, medical centers, and local, state, and federal governments.

Multisectoral partnerships that foster shared decision making and common goals can lead to innovative solutions like the warp-speed creation of the COVID-19 vaccination programs (24). Again, it is important to differentiate trials from actual vaccine rollouts. A systematic review of prevention and treatment clinical trials for COVID-19 in the US (25) estimated the representation of participants by sex, race, and ethnicity as compared to the US population with COVID-19 during that period. Underrepresented populations were generally underrepresented in these trials with the exception of Hispanics being over-represented in COVID-19 treatment trials. Similarly, initial vaccine uptake rates were dramatically lower in racial and ethnic minority groups although dedicated attention and resources has helped narrow gaps (26, 27).

## Discussion

The mission of precision medicine is to provide individualized treatment plans to patients, in part through the integration of advances in technology and genomics. Although precision medicine has been promoted for over a decade, too often the impacts of social determinants on health have been ignored, with patient management delivered in the absence of a clear understanding of the underlying social and cultural context. In addition to minority and ethnic populations being underrepresented in clinical research, older adults have also been characteristically underrepresented in clinical trials (28). To counter age-biases in research, the National Institute of Health now expects inclusion across the lifespan and explicitly require justification for age exclusions in its funded research portfolio (29). But nuance is also called for here, as chronological age categories are often distinct from biological/ functional statuses and have different treatment implications (30).

Hence, while precision medicine has promise, new paradigms are needed to ensure health equity for URM populations, and especially older adults. Everyone has a role to play in the roll-out of precision medicine and the translation of precision medicine into precision prevention. We urge federal and state governments, health care systems, biotechnology and pharma industries, clinicians and academics, patients, and community groups to become informed and to step up to the challenge of making precision medicine relevant to the health and wellbeing of all. This includes proactive steps such as having explicit goals to reduce extant health disparities, greater awareness of the importance of community-centric approaches, consideration of differentiated vs. homogeneous racial and sociodemographic categories, availability of diverse genomic data that is coordinated with a full range of health and social data, and improved provider-patient-community relationships to build needed trust in the health care system and the benefits of clinical research.

## Author contributions

MGO, OEA, KSR, PSS, and DVD helped to conceptualize the manuscript and provided a framework for furthering research at the intersection of health equity and precision medicine. MGO and OEA prepared first draft. KSR, PSS, and DVD added specific perspectives on precision medicine and genomics. All authors assisted in drafting the manuscript and reviewed and approved the manuscript.

## References

[1] U.S. Department of Health and Human Services. Healthy People 2030 (2022). Available online at: https://health.gov/healthypeople (accessed February 04, 2023).

[2] Silva P Dahlke DV Smith ML Charles W Gomez J Ory MG. An idealized clinicogenomic registry to engage underrepresented populations using innovative technology. J Pers Med. (2022) 12:713. 10.3390/jpm12050713 35629136 PMC9144063

[3] Snyder AA. Voice of the patient. In: The Patient and Health Care System: Perspectives on High-Quality Care. Cham: Springer Nature Switzerland (2020). p. 3–20. 10.1007/978-3-030-46567-4_1

[4] University of California San Francisco. Precision Medicine at UCSF (2015). Available online at: https://precisionmedicine.ucsf.edu/about (accessed February 04, 2023).

[5] *White House Precision Medicine Initiative*. The White House (2015). Available online at: https://obamawhitehouse.archives.gov/precisionmedicine (accessed February 04, 2023).

[6] US Food Drug Administration. Precision Medicine (2018). Available online at: https://www.fda.gov/medical-devices/in-vitro-diagnostics/precision-medicine (accessed February 04, 2023).

[7] Zambelli-Weiner A. The Three Roles Precision Medicine Plays in Advancing Health Equity. Healthcare Information Management Systems Society, Inc. (2021). Avaialble online at: https://www.himss.org/resources/three-roles-precision-medicine-plays-advancing-health-equity (accessed February 04, 2023).

[8] Mills MC Rahal C. A scientometric review of genome-wide association studies. Commun Biol. (2019) 2:9. 10.1038/s42003-018-0261-x 30623105 PMC6323052

[9] Turner BE Steinberg JR Weeks BT Rodriguez F Cullen MR. Race/ethnicity reporting and representation in US clinical trials: a cohort study. Lancet Reg Health Am. (2022) 11:100252. 10.1016/j.lana.2022.100252 35875251 PMC9302767

[10] Ferdinand A Kendal E. Equity in Precision Medicine. BioMed Central (2022). Available online at: https://www.biomedcentral.com/collections/precision (accessed February 04, 2023).

[11] Khoury MJ Bowen S Dotson WD Drzymalla E Green RF Goldstein R. Health equity in the implementation of genomics and precision medicine: a public health imperative. Genet Med. (2022) 24:1630–9. 10.1016/j.gim.2022.04.009 35482015 PMC9378460

[12] Bustamante CD De La Vega FM Burchard EG. Genomics for the world. Nature. (2011) 475:163–5. 10.1038/475163a 21753830 PMC3708540

[13] Callier SL. The use of racial categories in precision medicine research. Ethn Dis. (2019) 29(Suppl. 3):651–8. 10.18865/ed.29.S3.651 31889770 PMC6919973

[14] Gimbernat-Mayol J Dominguez Mantes A Bustamante CD Mas Montserrat D Ioannidis AG. Archetypal Analysis for population genetics. PLoS Comput Biol. (2022) 18:e1010301. 10.1371/journal.pcbi.1010301 36007005 PMC9451066

[15] Wilson JF Weale ME Smith AC Gratrix F Fletcher B Thomas MG. Population genetic structure of variable drug response. Nat Genet. (2001) 29:265–9. 10.1038/ng761 11685208

[16] Centers for Disease Control Prevention. The U.S. Public Health Service Syphilis Study at Tuskegee (2022). Available online at: https://www.cdc.gov/tuskegee/index.html (accessed February 04, 2023).

[17] Gamble VN. Under the shadow of Tuskegee: African Americans and health care. Am J Public Health. (1997) 87:1773–8. 10.2105/AJPH.87.11.1773 9366634 PMC1381160

[18] Davis JL Bynum SA Katz RV Buchanan K Green BL. Sociodemographic differences in fears and mistrust contributing to unwillingness to participate in cancer screenings. J Health Care Poor Underserv. (2012) 23:67. 10.1353/hpu.2012.0148 23124501 PMC3786428

[19] Rodríguez-Madera SL Varas-Díaz N Padilla M Grove K Rivera-Bustelo K Ramos J. The impact of Hurricane Maria on Puerto Rico's health system: post-disaster perceptions and experiences of health care providers and administrators. Glob Health Res Policy. (2021) 6:1–11. 10.1186/s41256-021-00228-w 34753513 PMC8577961

[20] St John JA Fox DJ Rosenthal EL Lee LK. Editorial: community health workers practice from recruitment to integration. Front Public Health. (2022) 10:841081. 10.3389/fpubh.2022.841081 35252104 PMC8891435

[21] Ramos IN Ramos KN Ramos KS. Driving the precision medicine highway: community health workers and patient navigators. J Transl Med. (2019) 17:85. 10.1186/s12967-019-1826-2 30876478 PMC6419796

[22] National Institutes of Health. All of US Research Program. Available online at: https://allofus.nih.gov/ (accessed February 04, 2023).

[23] Precision medicine needs an equity agenda. Nat Med. (2021) 27:737. 10.1038/s41591-021-01373-y 33990803

[24] United States Government Accounting Office,. Operation Warp Speed Accelerated COVID- 19 Vaccine Development Status Efforts to Address Manufacturing Challenges. United States Government Accountability Office (2021). Available online at: https://www.gao.gov/assets/gao-21-319.pdf (accessed February 04, 2023).

[25] Xiao H Vaidya R Liu F Chang X Xia X Unger JM. Sex, racial, and ethnic representation in COVID-19 clinical trials: a systematic review and meta-analysis. JAMA Intern Med. (2023) 183:50–60. 10.1001/jamainternmed.2022.5600 36469312 PMC9857303

[26] Ndugga N Hill L Artiga S Halder S. Latest Data on *COVID-19 Vaccinations by Race/Ethnicity Kaiser Family Foundation* (2022). Available online at: https://www.kff.org/coronavirus-covid-19/issue-brief/latest-data-on-covid-19-vaccinations-by-race-ethnicity/ (accessed February 04, 2023).

[27] COVID-19 Vaccine Equity for Racial Ethnic Minority Groups. Centers for Disease Control and Prevention (2022). Available online at: https://www.cdc.gov/coronavirus/2019-ncov/community/health-equity/vaccine-equity.html (accessed February 04, 2023).

[28] Herrera AP Snipes SA King DW Torres-Vigil I Goldberg DS Weinberg AD. Disparate inclusion of older adults in clinical trials: priorities and opportunities for policy and practice change. Am J Public Health. (2010) 100(Suppl. 1):S105–12. 10.2105/AJPH.2009.162982 20147682 PMC2837461

[29] Inclusion Across the Lifespan. National Institutes of Health (2019). Available online at: https://grants.nih.gov/policy/inclusion/lifespan.htm (accessed February 04, 2023).

[30] Maltoni R Ravaioli S Bronte G Mazza M Cerchione C Massa I. Chronological age or biological age: what drives the choice of adjuvant treatment in elderly breast cancer patients? Transl Oncol. (2022) 15:101300. 10.1016/j.tranon.2021.101300 34864401 PMC8640726

